# Co-circulation of the dengue with chikungunya virus during the 2013 outbreak in the southern part of Lao PDR

**DOI:** 10.1186/s41182-016-0020-y

**Published:** 2016-08-04

**Authors:** Viengvaly Phommanivong, Seiji Kanda, Takaki Shimono, Pheophet Lamaningao, Andrew Waleluma Darcy, Nobuyuki Mishima, Bounthanh Phaytanavanh, Toshimasa Nishiyama

**Affiliations:** 1 Department of Public Health, Kansai Medical University, 2-5-1, Shinmachi, Hirakata-shi, Osaka, 573-1010 Japan; 2 Champasak Provincial Health Department, Ministry of Health, Pakse, Champasak, Lao PDR

**Keywords:** Dengue virus, Chikungunya virus, RT-PCR, Co-infection, Outbreak, Phylogenetic analysis, Co-circulation

## Abstract

**Background:**

During the 2013 outbreak, 4638 infection cases and 32 deaths have been recorded in the southern part of Laos. In recent years, the chikungunya virus (CHIKV) emerged in the part of the country bordering Cambodia. Dengue virus (DENV) and CHIKV are transmitted by common mosquito vectors. Both diseases have similar clinical presentations; therefore, CHIKV infections might go undiagnosed in DENV-endemic areas. Thus, rapid detection and accurate diagnosis are crucial for differentiating between the two viruses (DENV and CHIKV). In this study, we demonstrated that CHIKV and two serotypes of DENV are circulating in Laos. In addition, we encountered patients that had been concurrently infected with multiple DENV serotypes or DENV and CHIKV.

**Methods:**

Plasma samples were collected from 40 patients with suspected DENV infections during an outbreak between July and August 2013. The reverse transcription polymerase chain reaction was performed to detect the four DENV serotypes and CHIKV using specific primers. Specifically, the complete envelope gene sequences of the viruses were sequenced and subjected to phylogenetic analysis.

**Results:**

Forty acute-phase plasma samples from patients with suspected dengue infections were tested for the presence of DENV viral RNA using molecular methods. Among the 40 samples, 14 samples were positive for DENV, 2 samples were positive for both viruses (DENV-2 and DENV-3), whereas DENV-1 and DENV-4 were not detected during the study period. We also encountered 10 samples that were positive for CHIKV. Of the 10 CHIKV-positive samples, 3 samples were co-infected by DENV-2, and 2 samples were co-infected by DENV-3. Phylogenetic analysis revealed that the 2013 dengue outbreak in Laos involved DENV-2 genotype Asian I and DENV-3 genotype II. Moreover, the Laotian CHIKV strains grouped together with those isolated during outbreaks on the Indian Ocean Islands within the East Central South African genotype.

**Conclusions:**

These findings revealed that two serotypes (DENV-2 and DENV-3) and CHIKV were detected. Furthermore, infection of multiple DENV serotypes and CHIKV was also observed in the 2013 dengue outbreak. This is the first documented evidence of co-infection with CHIKV and one of two DENV serotypes*.*

## Background

DF (dengue fever) is a mosquito-borne viral disease caused by the dengue virus (DENV), which belongs to the Flavivirus genus, *Flaviviridae* family, and has been categorized into four different serotypes (DENV-1 to DENV-4). It commonly occurs in tropical and subtropical regions [1]. The World Health Organization (WHO 2009) estimates that more than 50 million dengue infections occur yearly, resulting in half a million cases of dengue hemorrhagic fever (DHF) and 22,000 deaths, mainly among children. DENV is endemic in Southeast Asia, the Pacific, and the Americas [2]. However, in recent years, the hyperendemic circulation of all four dengue serotypes has been detected in Southeast Asian countries [3]. Other *Flavivirus* such as Japanese encephalitis (JE) is also endemic, occurring in Laos [4].

In Laos, dengue infections exhibit a cyclical pattern, i.e., they occur approximately every 2–5 years [5]. DENV serotypes responsible for such infections in Laos were first confirmed in 1994, and a case involving co-infection with two DENV serotypes was reported [6]. Since then, larger epidemics caused by all four serotypes have occurred [7, 8]. DENV-1 has emerged in several provinces and caused sporadic clinical cases in different areas of Laos between 2010 and 2011 [8]. The dominant circulating serotype subsequently switched from DENV-1 to DENV-3, and DENV-3 virus was the predominant DENV circulating in Laos at the end of June 2012 [7]. However, while some suspected cases of DENV infection were confirmed using laboratory detection, other cases of dengue infection were diagnosed based on clinical symptoms [9].

Chikungunya has been identified in more than 60 countries in Asia, Africa, Europe, the Americas, the Indian Ocean, and Pacific Islands [10]. In 2012, in a community survey, 31 % (16 of 52) cases of chikungunya virus (CHIKV) infection was recorded in the southern part of Laos [11]. The CHIKV is a member of the *Alphavirus* genus, which belongs to the *Togaviridae* family. Infection of CHIKV has similar clinical presentations with DENV and co-circulates in overlapping geographic regions; hence, it can be underdiagnosed in areas where the DENV-endemic occurs [10]. Few studies of the molecular epidemiology of serotypes/or genotypes of DENV and CHIKV were reported in Laos [7, 8, 11].

In the present study, the specimens were screened for the presence of DENV and CHIKV using the reverse transcription polymerase chain reaction (RT-PCR) during the 2013 outbreak of DF in southern Laos. Our results highlight that CHIKV and two serotypes of DENV are circulating in the southern part of Laos, which shares borders with Cambodia and Thailand. In addition, we encountered patients that had been concurrently co-infected with multiple DENV serotypes or DENV and CHIKV.

## Methods

### Study sites

**Fig. 1:**
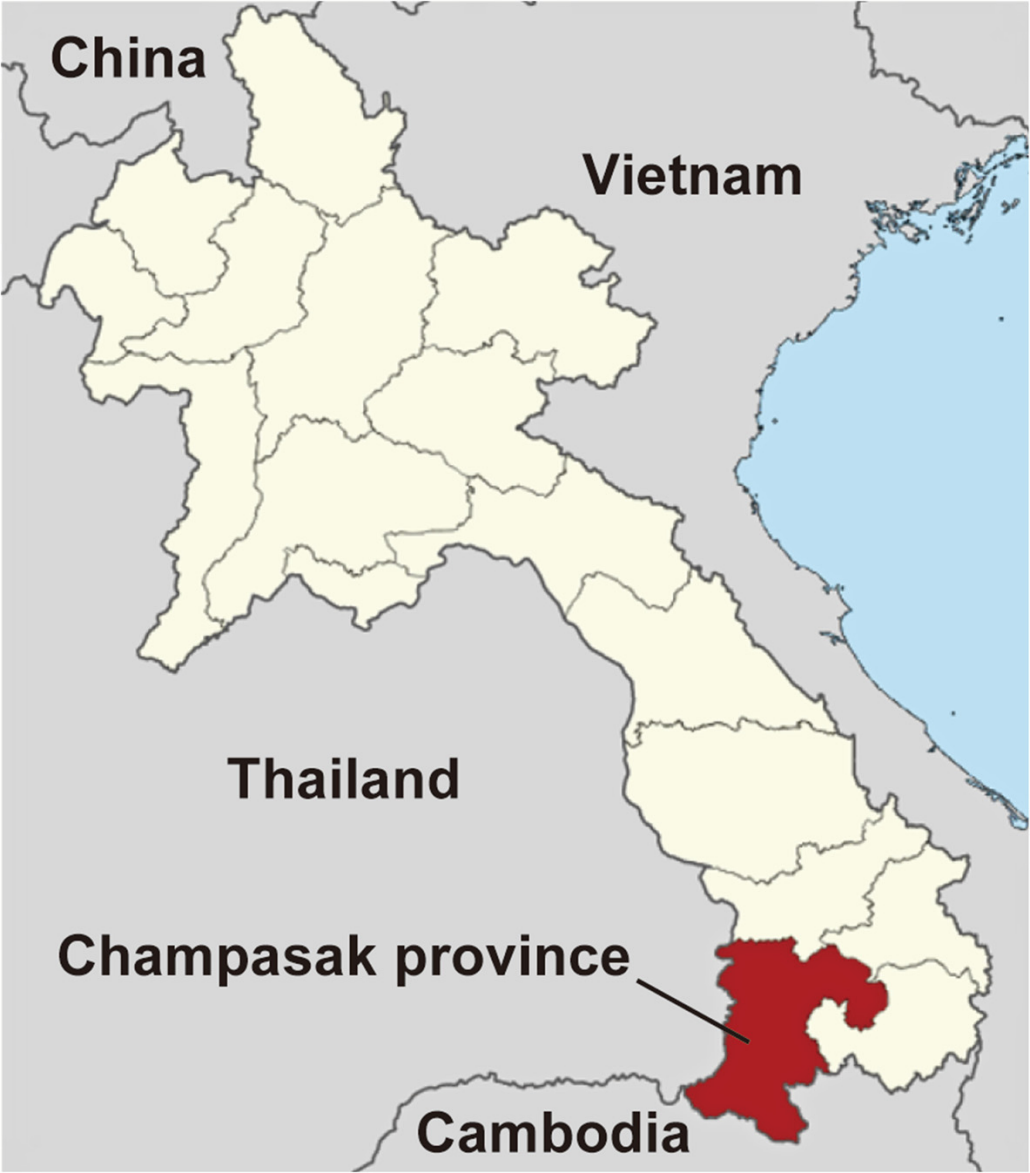
A map of the study area (Champasak Province, Lao PDR)

Champasak province (CPS) (610 km south of Vientiane capital) lies to the southwest in Laos (Fig. 1). It shares a border with Thailand to the west, Salavan, Sekong, and Attapeu provinces to the north and east, and Cambodia to the south. The Champasak hospital, a provincial hospital, is arranged in the third level of health services at the national level where there is inadequate laboratory facilities to diagnosis of infectious diseases.

### Clinical characterization of patients and sample collection

Forty hospitalized patients and 3 additional cases (1 case from Oudomsay province and 2 cases from Vientiane capital) were investigated during the outbreak of DF/DHF from the end of July to the beginning of August 2013. Forty patients, aged 5 to 65 years presented with acute DENV infection at days 1–6 after the onset of fever with two more of the following symptoms: headache, myalgia, arthralgia, skin rash, and hemorrhage. All of these 40 patients were diagnosed with DENV infection. The history of their illness and complete blood counts: white blood cells (WBC), platelet counts (PLT), and hematocrit (HCT), were obtained from a physician at the Champasak hospital.

A total of 8–10 ml of whole blood samples are collected in tubes that contained EDTA as an anticoagulant. Plasma samples were separated and preserved in an RNA Shield*™* reagent (Zymo Research) that could protect from RNA degradation. These specimens were then transferred to the Laboratory of Public Health department, Kansai Medical University, Japan.

### Laboratory procedures

The plasma samples were separated from the patients’ whole blood by centrifugation at 1000×*g* for 5 min at 4 °C. A total of 200–500 μl of plasma samples were directly used for the viral RNA extraction and RT-PCR. The remaining plasma specimens were kept at −20 °C prior to testing and were stored at −80 °C until further use.

### RNA extraction and PCR

Total RNA was extracted from patient’s plasma sample using TRIzol® reagent (Invitrogen Inc.), according to the manufacturer’s protocol with the following modifications. Then, the extracted RNA was used to synthesize first-strand cDNA with random primers and reverse transcriptase (ReverTra Ace®: Toyobo) for 1 h at 42 °C [12]. In the PCR analysis, the cDNA was used as a template and amplified using serotype-specific primers for serotypes D1 to D4 of DENV according to the method of Lanciotti et al. [13] or a specific primer for CHIKV [14]. The general PCR conditions were as follows: 94 **°**C for 2 min, 98 °C for 10 s, and 54–62 °C for 30 s for 35–40 cycles. After their amplification, the PCR products were electrophoresed and visualized by staining 1.5 % agarose gel with ethidium bromide, and specific bands were visualized with an ultraviolet transilluminator.

### Sequencing of the envelope (E) gene and E1 gene

In order to identify the genotypes of DENV and CHIKV, we tried to analyze the sequences of the DENV-2, DENV-3, and CHIKV isolates detected during the screening process described above. PCR was performed by using cDNA derived from the DENV-2-, DENV-3-, or CHIKV-positive patients’ samples as a template and a primer pair for each target region to amplify the complete envelope (E) gene of DENV and E1 envelope glycoprotein gene of CHIKV. The following sets of specific primers for DENV-2 (Den2-911F 5′-TGACRG CTGTCGCTCCTTCA-3′, Den2-2444R 5′-CARCTCACAAYGCAACCACTATC-3′, 1485 bp), DENV-3 (Den3-815F 5′-GCCCTTAGGCACCCAGGGTT-3′, Den3-1752R 5′-CCCGCGAAAATGCTTGTGC-3′, Den3-1398F 5′-CGCAAGGAG TCACGGCTGAG-3′, Den3-2539R 5′-GCCTGCAATGGCTGTTGCC-3′, 1479 bp) [7], and CHIKV (Chik E1Fseq1 5′-GCTCCGCGTCCTTTACC-3′, Chik E1RSeq1 5′-ATGGCGACGCCCCCAAAGTC, 540 bp) were used for the PCR amplification. The PCR amplicons were directly sequenced using the BigDye® Terminator v3.1 cycle sequencing kit (Applied Biosystems). The sequencing was performed using the following conditions: 96 °C for 1 min followed by 35 cycles of 96 °C for 10 s, 50 °C for 5 s, and 60 °C for 4 min. Sequence chromatograms for both strands were obtained using an ABI3730XL automated sequence analyzer (Applied Biosystems).

### Phylogenetic analysis of DENV and CHIKV

The complete nucleotide sequences of the E gene of the Laotian DENV-2 (1485 bp) and DENV-3 (1479 bp) strains, and the partial nucleotide sequences of the E1 gene of CHIKV (540 bp) were aligned using ClustalW [15]. A phylogenetic tree was constructed using the maximum likelihood (ML) method. The ML analysis was performed using the General Time Reversible (GTR) model with a gamma distribution, and the proportion of invariable sites (I) was estimated by MEGA v5.2 (www.megasoftware.net) [16]. The reliability of the analysis was evaluated in a bootstrap test with 10,000 replications. Representative strains of the DENV-1 and DENV-3 serotypes were used as the outgroup taxon for the DENV-3 and DENV-2 tree, respectively. The sequence of the O’nyong-nyong virus, strain IPD A234 (GenBank accession number: NC001512 and AF192890), was used as an outgroup for the CHIKV tree [17]. Sequences of all Laotian DENV and CHIKV are deposited in the DNA Data Bank of Japan (DDBJ) under accession number LC147056-LC147057 for DENV-2, LC147058-LC147061 for DENV-3, and LC147062-LC147064 for CHIKV, respectively.

### Ethics statement

This study was approved (No. 276/NECHR) by the National Ethics Committee for Health Research, Ministry of Health, Lao PDR, and the Institutional Review Board of Kansai Medical University (reference no.1430). Informed consent was obtained from each participant, as well as parental permission for children involved in the research.

## Results

### Clinical features

All of the plasma samples were collected from patients with suspected DENV infections that were treated at the Champasak hospital during an outbreak of DF. Forty subjects were enrolled (13 in the 5–15 years age group, 23 in the 16–45 years age group, and 4 in the 46–65 years age group), and 22 (55 %) of them were female. The median age of the patients was 20.50 years (range 5–65 ).

As shown in Table 1, all of the patients developed a fever (days 1–6) and produced positive results in the tourniquet test. Nearly all of the patients (97.5 %) experienced headaches during their hospitalization. Muscle pain was present in 87.5 % of patients, and joint pain (70 %) and retro-orbital pain (72.5 %) were also common. Digestive problems were observed in 17 (42.5 %) patients. The patients’ other symptoms included chills (17.5 %), skin rash (15 %), bleeding from the nose or gums (5 %), petechiae (5 %), and bleeding that occurred within 8 days of onset (2.5 %). Seventy-nine percent of the patients exhibited lower white blood cell counts (leukopenia <5000/mm^3^). Thrombocytopenia (<100,000/mm^3^) was observed in 34 % of cases, and 23 % of patients were presented with increases in their HCT levels of >20 % compared with the baseline. There were no deaths during the study period.

**Table 1:** Clinical features of hospitalized patients (*N* = 40)

Symptoms and clinical tests	No. of patients	%
Symptoms		
Fever	40	100
Headache	39	97.5
Retro-orbital pain (eye pain)	29	72.5
Digestive problems (nausea/vomiting)	17	42.5
Muscle pain (myalgia)	35	87.5
Join pain (arthralgia)	28	70
Chills	7	17.5
Skin rash	6	15
Petechiae	2	5
Bleeding nose or gum	2	5
Bleeding within 8 days	1	2.5
Clinical tests		
Tourniquet test	40	100
Leukopenia (<5000/mm3)	30	78.9
Thrombocytopenia (<100,000/mm3)	13	34.2
Elevated hematocrit (>20 % increased)	9	23.1

### Screening of clinical samples by PCR

Detection and typing of the four DENV serotypes and CHIKV in plasma samples by PCR assay using specific primers for DENV serotypes 1 to 4 and CHIKV.

In the results of the 40 specimens, 7 (17.5 %) and 5 (12.5 %) were found to be positive for DENV-2 and DENV-3, respectively. However, DENV-1 and DENV-4 were not detected in the present study. Furthermore, DENV-2 and DENV-3 co-infection was detected in 2 (5 %) samples. Moreover, CHIKV was also detected in 10 samples (25 %). Of the 10 CHIKV-positive cases, 3 samples were co-infected by DENV-2 and 3 samples co-infected by DENV-3, respectively. The sequences of these PCR products from all positive samples were also confirmed by sequencing analysis.

### DNA sequencing analysis

Serotypes/genotypes were determined by PCR and/or sequencing analysis using forward and reverse primers of the complete envelope gene of DENV-2 (Den2-911F and Den2-2444R, 1485 bp) and DENV-3 (Den3-815F and Den3-1752R; Den3-1398F and Den3-2539R, 1479 bp), and partial E1 gene of CHIKV (Chik E1Fseq1 and Chik E1RSeq1, 540 bp). Entire gene sequences of two DENV-2, four DENV-3, and partial gene sequences of three CHIKV were then analyzed by phylogenetic analysis. The results showed that the percentage of similar among the two DENV-2 was 99 %, four DENV-3 ranged from 90 to 97 %, CHIKV ranged from 62 to 67 % when those compared to each other and to strains representative of the different serotypes/genotypes available on GenBank.

### Phylogenetic analysis of DENV-2

**Fig. 2:**
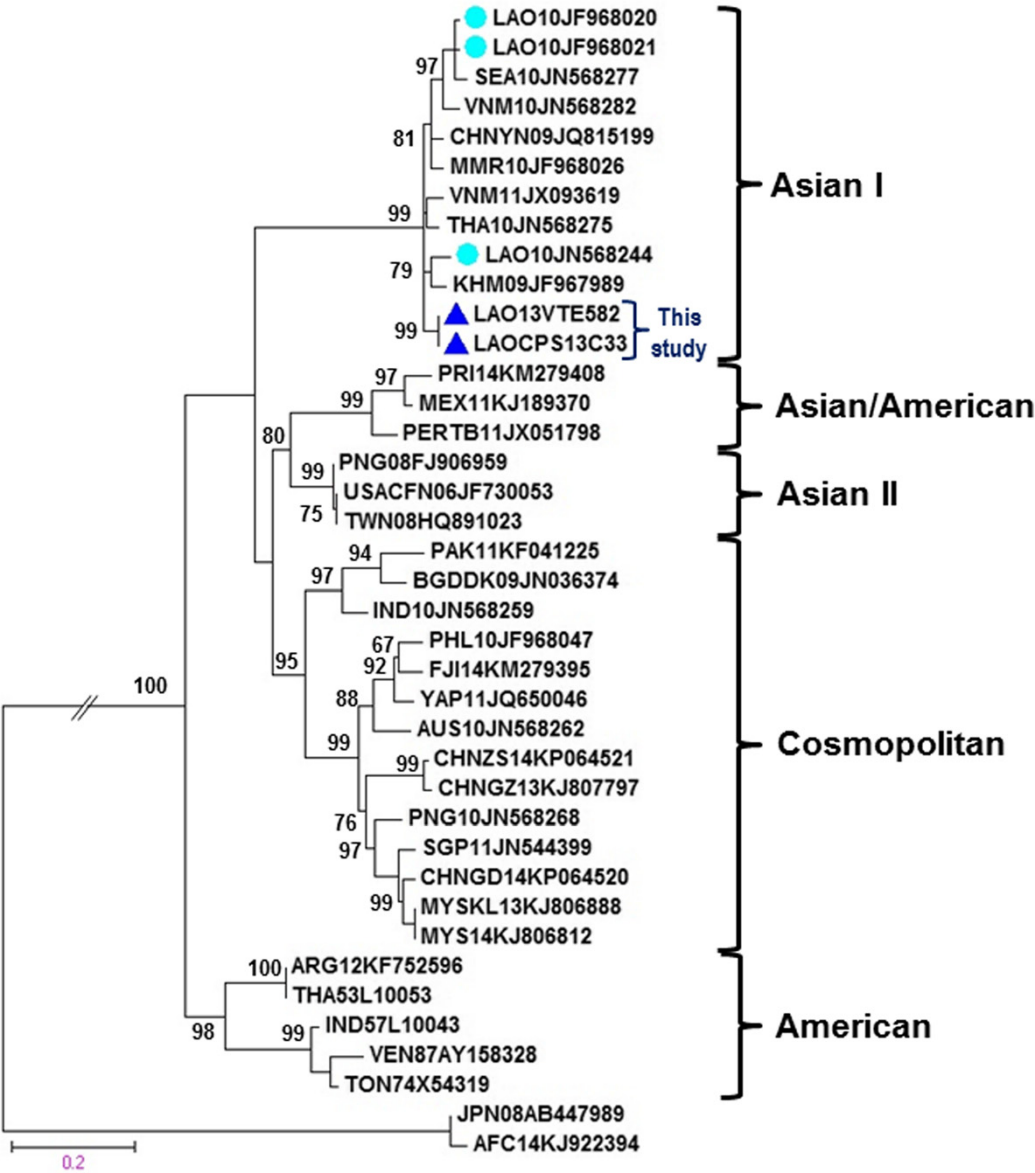
A maximum likelihood tree constructed based on the complete envelope gene sequence of DENV-2. Each DENV-2 isolate is shown together with its country of origin followed by two digits, which indicate the year in which it was isolated, and its GenBank accession number. Two representative strains that were isolated in the present study are indicated by *filled triangles*. Bootstrap values of <60 % are not shown. The scale bar indicates the mean number of nucleotide substitutions per site

The complete E gene sequences of two distinct DENV isolates (LAO13VTE582 and LAOCPS13C33) from the 2013 outbreak were determined and compared with sequences of 37 representative DENV-2 strains of each genotype published in GenBank. Two strains of DENV-2 from Laos viruses were closely related each other and belonged to genotype Asian I (Fig. 2). The genotype Asian I consists of viruses mainly from Southeast Asia, including Thailand, Cambodia, Vietnam, China, and Myanmar. No Asian II genotype and Asian/America genotype strains were found during dengue outbreak in Laos 2013.

### Phylogenetic analysis of DENV-3

**Fig. 3:**
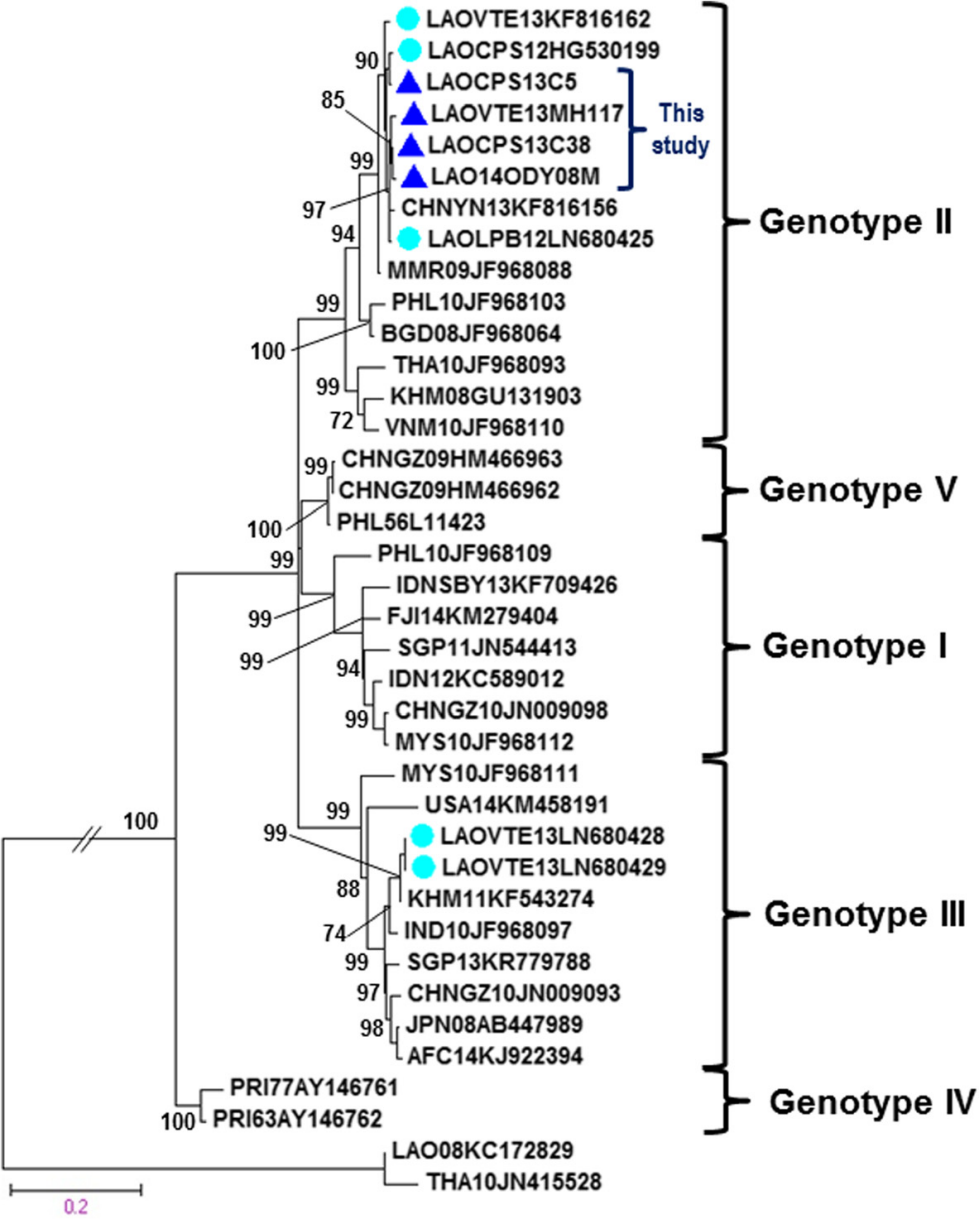
A maximum likelihood tree constructed based on the complete envelope gene sequence of DENV-3. Each DENV-3 isolate is shown together with its country of origin followed by two digits, which indicate the year in which it was isolated and its GenBank accession number. Four representative strains that were isolated in the present study are indicated by *filled triangles*. Bootstrap values of <60 % are not shown. The scale bar indicates the mean number of nucleotide substitutions per site

The DENV-3 strains isolated in the current study and previously isolated DENV-3 strains from other provinces of Laos (Laungprabang, Oudomsay, and Champasak) and Vientiane were compared with sequences of 34 representative DENV-3 strains of each genotype obtained from GenBank database. Sequences of four strains for DENV-3 from Laos were grouped together within genotype II (Fig. 3). The genotype II of DENV-3 is common in Southeast Asian countries and clusters within the viral strains from China, Myanmar, the Philippines, Bangladesh, Thailand, Cambodia, and Vietnam. The additional DENV-3 isolated in Vientiane in 2013 (LAOVTE13LN680428 and LAOVTE13LN680428) [7] belong to genotype III (Fig. 3). No genotype I and genotypes III strains were found in the study period.

### Phylogenetic analysis of CHIKV

**Fig. 4:**
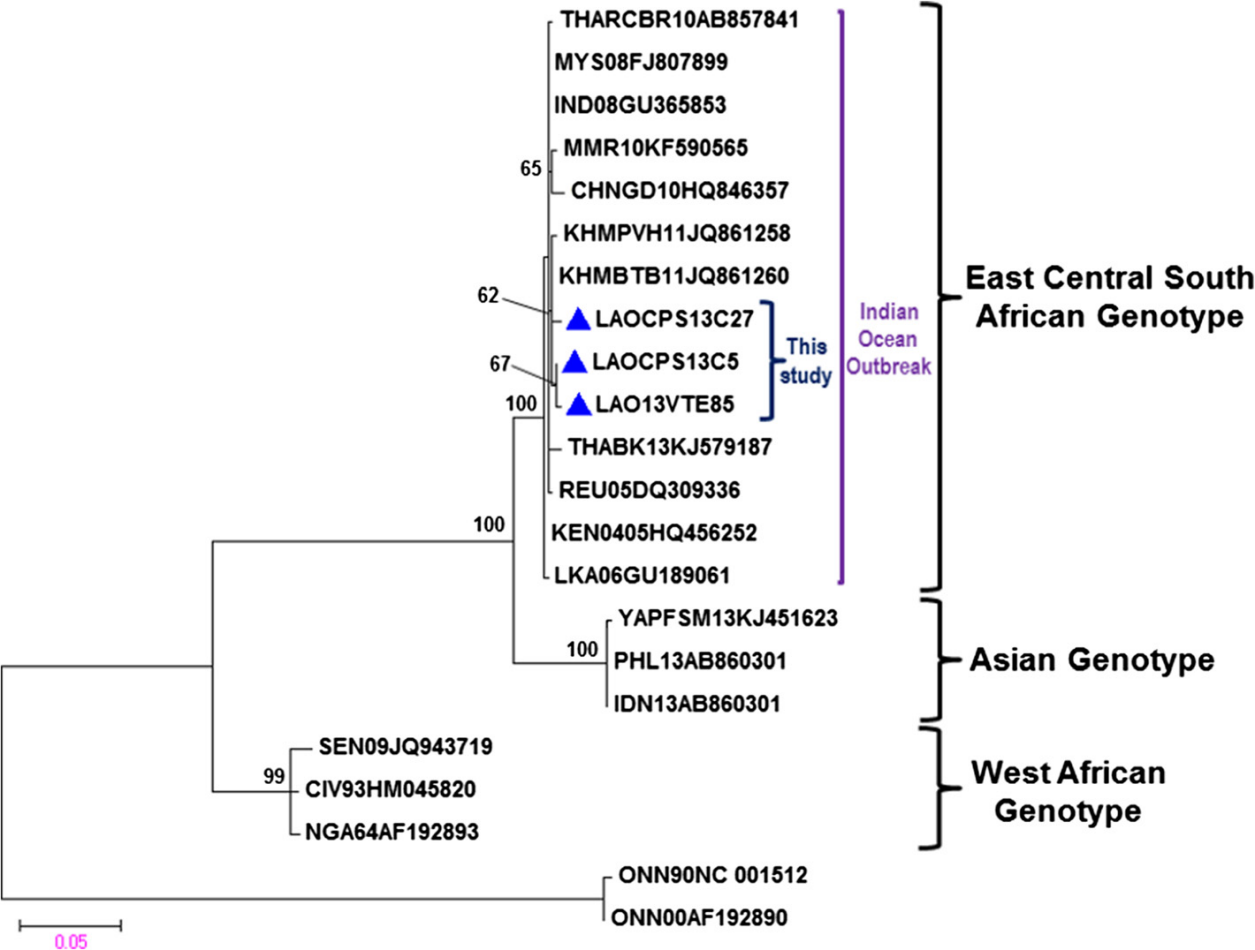
A maximum likelihood tree constructed based on the partial envelope (E1) gene sequence of CHIKV. Each CHIKV isolate is shown together with its country of origin followed by two digits, which indicate the year in which it was isolated and its GenBank accession number. Three representative strains that were isolated in the present study are indicated by *filled triangles*. Bootstrap values of <60 % are not shown. The *scale bar* indicates the mean number of nucleotide substitutions per site

Analysis of the partial E1 gene sequences of 19 representative strains of each genotype of CHIKV published in GenBank, including sequences of three representative strains of CHIKV from Laos demonstrated that all CHIKV strains from the present study were closely related to each other and other viruses from Cambodia (isolated in 2011) [18]. All study sequences clustered together with the causative CHIKV strains isolated during an epidemic in the Indian Ocean Islands and belonged to the East Central South African genotype (ECSA). The ECSA genotype consists of viral strains from Southeast Asia and other countries, including Reunion Island and Kenya (Fig. 4).

## Discussion

In 2013, Laos experienced a major DF/DHF outbreak presented with nearly 50,000 dengue cases and 92 deaths (MOH, 2013). Because CHIKV infection has similar clinical features with DENV infection and co-circulates in overlapping geographic distributions, therefore, CHIKV may be misdiagnosed in areas where DENV endemic occur [10]. During dengue fever outbreak, the Lao medical doctor only diagnosed the dengue infections among patients. Consequently, we want to identify that these patients are really infected by dengue virus or other infectious disease. The present study showed that fever, headache, retro-orbital pain, a positive tourniquet test, and body and joint pain are common symptoms in patients that have been infected with DENV. Additionally, our data also revealed that arthralgia (joint pain) and skin rash were the most common symptoms found in CHIKV-infected patients (data not shown), and similar findings were reported by Ali et al.[19].

In our study, a molecular screening specific for both DENV and CHIKV infections was performed on 40 acute-phase plasma samples collected from patients with suspected dengue infection in southern Laos during an outbreak between July and August 2013. DENV was detected by PCR in 30 % and CHIKV in 12.5 % of samples. Two samples (5 %) were co-infected by both viruses (DENV-2 and DENV-3), and five samples (12.5 %) were co-infected by DENV and CHIKV, respectively. Although the enrolled patients included five cases that were suffering from DHF, none of the patients died, and no cases of DENV-1 or DENV-4 were found during the study period. In our analysis of 40 samples, 52.5 % were found to be dengue-negative by RT-PCR. These samples might not have been collected during the acute phase of the infection (plasma viremia reduction) [20].

In Laos, the dominance serotype changes from year to year since 2010. DENV-1 was dominant in 2010 and 2011; DENV-3 was dominant in 2012 followed by DENV-2, according to the National Dengue surveillance, Lao PDR [8]. Our findings indicated that both DENV-2 (17.5 %) and DENV-3 (12.5 %) were dominant serotypes circulating in southern Laos in 2013. In addition, other researchers reported that DENV-3 (94 %) was dominant, followed by DENV-2 (3 %) circulating virus in Vientiane capital, whereas few cases of DENV-1 and DENV-4 (ranged from <1 to <6 %) have been recorded from May 2012 to December 2013 [7]. That corresponds with our data; DENV-1 and DENV-4 were not detected. Concurrent infection by multiple DENV serotypes (DENV-2 and DENV-3) was identified during the 2013 dengue outbreak in Laos. Furthermore, co-circulation of DENV-2 (38.7 %) and DENV-3 (29.3 %) were also reported in Thailand during dengue outbreak from 2004 to 2010 [21]. These findings suggested that DENV serotype 2 and 3 may have remained viruses in the circulation in these areas for a long time or they may have been introduced from a neighboring country such as Thailand. Geographically, Laos is located nearby Thailand compared with other countries in Southeast Asia. With the increased movement and/or migration of infected people within and between countries, hyperendemicity (the co-circulation of multiple DENV serotypes) may be occurred [22].

The first case of dual infection with DENV-1 and DENV-2 was a resident in Vientiane, the capital of Laos, who was presented with mild symptoms of dengue, which were not severe enough to require admission [6]. Since then, there have been no further reports of dual DENV infections in Laos. According to the data obtained in the present study, we also found that the co-infected patients were more likely to present the DHF including, fever, digestive trouble, skin rash, a positive tourniquet test, leukopenia, and bleeding; these patients needed admission to hospital during their illness.

We determined the genotypes of the isolated DENV-2 and DENV-3 viruses via phylogenetic analyses of their complete E gene sequences. DENV-2 is categorized into five genotypes: cosmopolitan, Asian-I, Asian-II, Asian-American, and American [23].

Based on complete E gene sequences, DENV-2 has been divided into five genotypes: Cosmopolitan, Asian-I, Asian-II, Asian-American, and American [23]. The Laotian DENV-2 were collected in the 2013 outbreak from different localities in Laos (610 km South (Champasak province)–Central (Vientiane capital)). Sequences of these two viruses strains of DENV-2 were closely related within genotype Asian I (Fig. 2). The genotype Asian I of DENV-2 isolates from Laos in 21010 and 2013 grouped together with viruses from Southeast Asian countries, including Cambodia (2009), Thailand (2010), Vietnam (2010 and 2011), Myanmar (2010), China (2009), Southeast Asia (2010), and Laos (2010) [24, 25]. The genotype Asian I found in the current study and those from Southeast Asian countries formed a monophyletic relationship with very high support values greater than 98 % are shown. We suggested that the genotype Asian I of DENV-2 has remained in dominant circulation in Laos for a long time since 2010 until an outbreak in 2013. The genotype Asian I of DENV-2 is also the predominant genotype circulating in many parts of Southeast Asia, except Malaysia, Singapore, Indonesia, and the Philippines [23].

Among the five genotypes of DENV-3 (I–V) [26], sequences of DENV-3 strains from the 2013 outbreak, together with other Laotian sequences collected from Laungprabang, Oudomsay, and Champasak provinces and Vientiane capital were grouped into the same cluster within genotype II (Fig. 3). The Laotian DENV-3 genotype II isolates were most closely related to those isolated from China (2013), Myanmar (2009), Bangladesh (2008), the Philippines (2010), Thailand (2010), Cambodia (2008), and Vietnam (2010) [25, 27]. All of the Laotian DENV-3 genotype II viruses obtained in this study and sequences from other Southeast Asian countries formed a monophyletic relationship with very high values bootstrap support (>98 %). We suggested that they had a single origin and have been circulating in Lao PDR for a long time. Two different genotypes of DENV-3 (genotype II and III) have been reported to have co-circulated in Laos in 2013 [7]. Even though two studies have been implemented in the same year, the findings are not the same. Although our sample size is small, the analysis presented in this study suggested that DENV-3 genotype II is circulating in the southern parts of Laos and has also invaded other parts of the country. Moreover, DENV-3 genotype II is the dominant circulating genotype in many countries in Southeast Asia [25].

Despite the small number of reported cases at the National dengue surveillance in the Lao PDR, and the fact that our study could only identify that two (5 %) cases of concurrent co-infection of DENV serotypes 2 and 3 were observed, Lardo et al. reported that concurrent infections of dengue viruses 2 and 3 have been proposed as one of contributing factors to severe dengue [28]. In the present study, it is difficult to conclude that a co-infected patient with two serotypes (i.e., DENV-2 and DENV-3) became afflicted with a more severe form of dengue (DHF/DSS) because of only two cases were experienced. Moreover, we did not have enough information about their clinical symptoms during hospital admission. In addition, the relationship between concurrent infections and severe forms of dengue (DHF/DSS) requires further study.

On the other hand, the current chikungunya epidemic in Southeast Asia is being driven by the appearance of a strain of CHIKV that originated in Africa [29] and spread to Asian countries such as Cambodia [18] and Thailand [30]. At present, CHIKV is known to be circulating in southern Laos [11] and is currently spreading to other regions of the country. During the 2013 outbreak of DENV in Laos examined in this study, we also found patients that had been infected with CHIKV. In fact, CHIKV-positive patients accounted for 25 % (10/40) of patients and 12.5 % (5/40) of the patients were co-infected with DENV-2 or DENV-3. Other studies have already recorded a high proportion of double infected cases with CHIKV and DENV (29 % from New Delhi, India, 12.4 % from West Bengal, India, and 37 cases from Gabon) [31, 32]. Detection of double infection of CHIKV and DENV in this study demonstrated the probability that many chikungunya cases may go misdiagnosed in areas where two viruses coexist [10]. In Laos, a diagnosis of dengue and chikungunya infection was based on patient’s clinical symptoms and in general samples were not checked by serological test such as a rapid test. In this study, we did not perform the virus isolation from samples.

Phylogenetic analysis divided CHIKV isolates into three distinct genotypes based on their geographic origins: the West African (WAf) genotype, East/Central/South African (ECSA) genotype, and Asian genotype [33]. Our findings demonstrated that the partial E1 gene sequences of the Laotian CHIKV strains clustered together with homologous strains from Indian Ocean CHIKV outbreaks within the ECSA genotype. All of these Laotian CHIKV strains were closely related to the CHIIKV strains that caused outbreaks in Cambodia, but not high bootstrap support values below 70 (Fig. 4) [18] and clustered together with other isolates from recent outbreaks in Asian countries (Thailand, Myanmar, China, Cambodia, Malaysia, Sri Lanka, and India) [18, 30, 34]. A high degree of sequence similarity between the Laotian and Cambodian strains and the fact that the Cambodian CHIKV outbreak occurred in 2011 where sharing borders with southern Laos and data from community survey [11], we suggested that CHIKV ECSA genotype is still endemic or is continuously reintroduced to the area and has invaded various regions of Laos.

## Conclusions

Dengue is still a prevalent mosquito-borne disease in Laos. Molecular detection and serotyping of dengue and chikungunya were carried out on acute-phase plasma samples that were collected during the 2013 dengue fever outbreak from Laos. Our data suggested that the identification of concurrent infection with two serotypes (DENV-2 and DENV-3) and co-infections with CHIKV and two DENV serotypes have been confirmed during the 2013 outbreak. Furthermore, our study indicated that the occurrence of DENV and CHIKV co-infections occurred in areas where these two viruses co-circulated. This is the first study to report on patients that had been co-infected with CHIKV and one of two DENV serotypes in Laos. These findings from our study will be helpful in the mitigation of priority actions such as improving surveillance and timely intervention to present and future outbreak threats.
